# Implementation of Point-of-Care Ultrasound (PoCUS) in Geisinger Health System

**DOI:** 10.7759/cureus.71621

**Published:** 2024-10-16

**Authors:** William Adams, Ene C Chukwuemeka, Calvin Kiniale, Jennifer Bekker, Hugh Johnson, Mathangi Rajaram-Gilkes

**Affiliations:** 1 Medical Education, Geisinger Commonwealth School of Medicine, Scranton, USA

**Keywords:** ct vs pocus, pocus, pocus in outpatient setting, pocus integration, point-of-care-ultrasound

## Abstract

Background

Point-of-care ultrasound (PoCUS) is an imaging modality with many inherent benefits, such as increased patient satisfaction, cost savings, and elimination of delays in diagnosis. The popularity of this bedside imaging technique has increased in recent years, and its scope of use for diagnostics and procedures has expanded in many specialties.

Research question

Can increased implementation of PoCUS within the Geisinger Health System (GHS) reduce the need for other more expensive, time-consuming, and potentially harmful imaging techniques?

Methods

Investigations were carried out on the uses of PoCUS established in the literature as a bedside diagnostic tool for specific pathologies such as pneumonia, nephrolithiasis, and various fractures. The SlicerDicer tool from Epic was then used to quantify diagnostic tests that could be replaced with PoCUS. Data collection focused on the number of other imaging modalities (CT, MRI, X-ray) that could be substituted with PoCUS. Data regarding the existing use of PoCUS within GHS could not be collected due to the limitations of SlicerDicer.

Results

Between January 1, 2019, and December 31, 2023, 121,057 imaging studies, including 38,838 CT scans, were recorded in SlicerDicer, where PoCUS could be implemented as an alternative diagnostic tool for specific pathologies. The largest portion of PoCUS-replaceable scans was chest X-ray at 61,684, followed by CT of the abdomen and pelvis at 36,204 scans in the five-year study period.

Conclusions

Our review of SlicerDicer data from GHS revealed substantial numbers of imaging scans that could be substituted with PoCUS. Expanding the use of PoCUS within GHS would be advantageous to both patients and providers, and we recommend that providers take advantage of opportunities to incorporate PoCUS into their clinical practice.

## Introduction

The purpose of our study is to identify instances within the Geisinger Health System (GHS), a large health-based system located in Northeast and Central Pennsylvania, where point-of-care ultrasound (PoCUS) is used to diagnose patients as an alternative method to other expensive or time-consuming radiological testing modalities. PoCUS has been increasing in popularity over the years as it is a quick and efficient way for clinicians to obtain imaging necessary to rule in or out conditions immediately at the bedside [1]. It was traditionally used in times when complex imaging was unavailable; however, when used in combination with a medical history and physical exam, it is a diagnostic test of improved speed and accuracy that can prevent delays between disease presentation and effective treatment [2]. Created initially to detect free intraperitoneal fluid in patients after trauma, it has since increased in popularity over the past 10 years to be additionally used in the emergency department and critical care settings such as abdominal or gynecological urgencies [2,3]. 

PoCUS has shown advantages such as improved portability, speed, and accessibility over other imaging modalities [1,3] and has become commercially available with the new implementation of pocket probes that work with various computers and mobile devices to capture ultrasound imaging and easily share it with others [4]. Newer probes can emulate different kinds of transducers depending on the tissue viewed, expanding on the potential disease conditions PoCUS can capture [4]. Other imaging modalities such as MRI and CT can take hours and require a facility with trained faculty to properly operate the large machines as well as a provider who can create a written report of the results. Instead, PoCUS provides valuable diagnostic information immediately and can be accurately read by the provider carrying out the investigation [1]. Compared to other imaging modalities, the continued improvement of PoCUS, with its ability for a quick and accurate diagnosis in emergencies, highlights its importance and potential for future expansion into other areas of medicine. 

GHS comprises 10 hospital campuses and 133 outpatient clinics across 67 counties in Northeast Pennsylvania. With 25,000 employees, including over 1700 physicians and an affiliated medical school, Geisinger Commonwealth School of Medicine (GHS) is a regional leader in healthcare innovation [5]. Our data from SlicerDicer reveals significant opportunities for using PoCUS within the GHS for a wide variety of complaints. For the purpose of this research, the authors of this article retrospectively identified 121,057 imaging scans of various modalities (e.g., X-ray, MRI, and CT) previously stored within a centralized database (described in the Materials and Methods section) across five years in GHS that could have been replaced with PoCUS. Of these, 38,838 were CT scans. Even if only a small portion of these scans are replaced with PoCUS, it will relieve a significant burden on patients, as well as hospitals and radiology departments across the health system. 

We recommend that providers who are appropriately trained begin maximizing the use of PoCUS where it is proven as an accurate diagnostic tool. We further recommend that clinicians unfamiliar with PoCUS undergo specific training to take advantage of this widely beneficial imaging modality.

## Materials and methods

**Figure 1:**
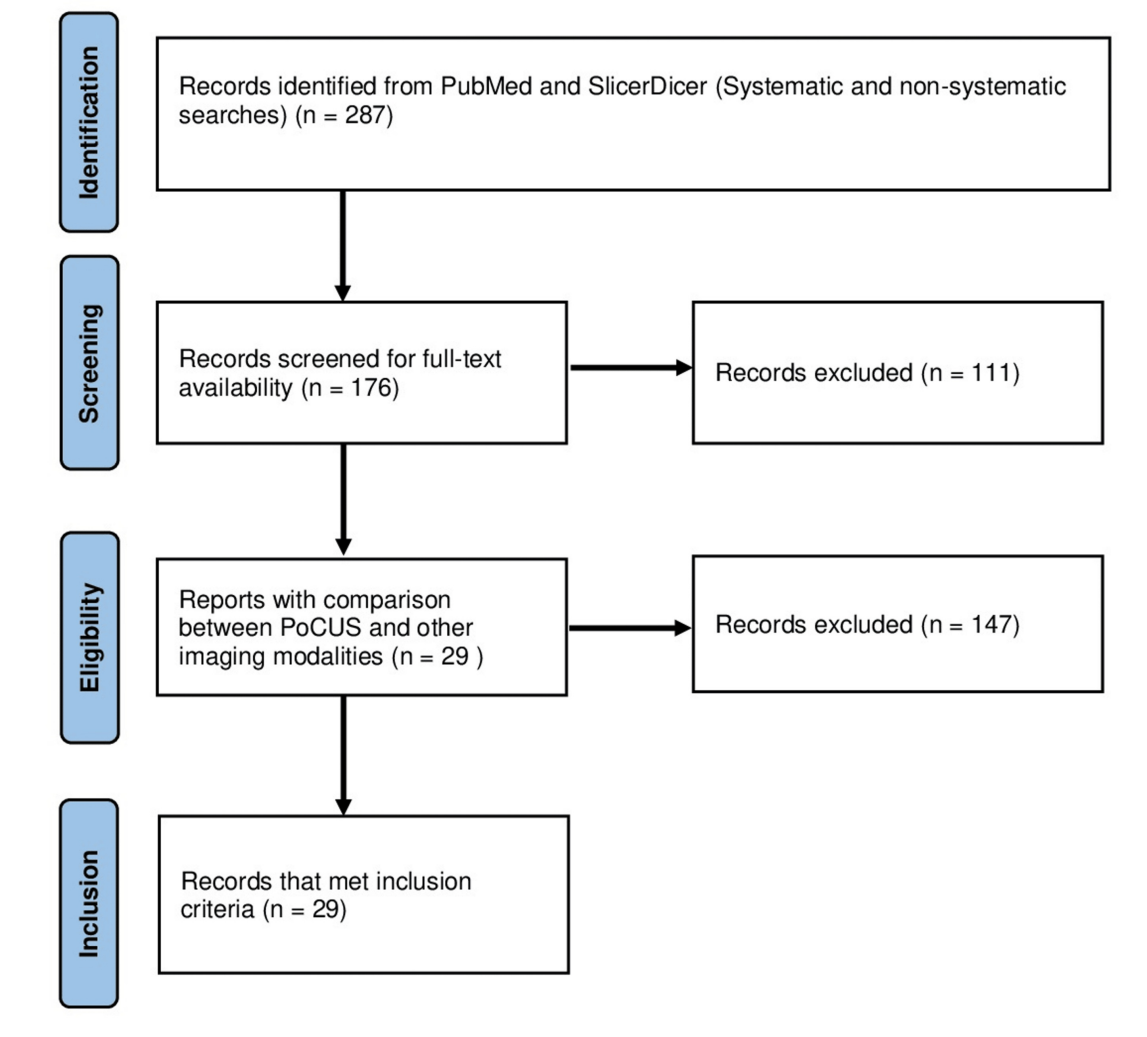
PRISMA chart for literature search

The two main methods of obtaining information were a literature search of the PubMed database and SlicerDicer data collection. A total of 287 articles were identified from PubMed through initial systematic and non-systematic searches. Of these, 176 had full-text availability. Articles were then reviewed by reading the title and abstract, and 29 were selected for final inclusion. Figure 1 shows the total number of articles at each stage of the selection process.

When reviewing each paper, the first criterion for inclusion was a clear comparison between PoCUS and another imaging modality such as CT or X-ray. Also required was a setting of primary care, emergency medicine, or orthopedics. Special attention was paid to PoCUS scans for musculoskeletal diagnoses such as fractures and ligament injuries, studies with additional measurements of patient benefits or time-to-diagnosis, and studies where ultrasound was substituted for a substantially more expensive test such as CT or MRI.

The excluded papers included case reports, those specific to COVID-19, those in a setting outside of primary care, emergency medicine, or orthopedics, and uses of PoCUS for a disease or condition unlikely to be encountered in Northeast Pennsylvania. These exclusions were made via PubMed search criteria. Most manual exclusions were made because there was not a clear comparison between PoCUS and another imaging modality, with 147 papers being excluded for this reason. Papers that were duplicates in supporting the same uses of PoCUS and had no measured additional benefits such as mortality reduction were also excluded. Sixteen papers were excluded for this reason.

SlicerDicer is part of Epic’s Cogito analytics module. It is a software program with broad applications such as quality improvement, population health monitoring, and comparing various patient cohorts [6]. SlicerDicer has access through Epic to a wide-ranging record of deidentified patient information that records the number of procedures, such as imaging studies, surgeries, and injections. It can also analyze types of office visits, hospital length-of-stay, patient demographics, diagnoses, and chief complaints. Various criteria can be added as “slices” to generate a cross-sectional result showing the number of patients who meet a specific set of criteria, e.g., the number of patients over 65 who attended an outpatient clinic visit in the past three months. All of this data is based on records from the Epic electronic health records (EHR). We had access to data from the Geisinger Health System for over five million patients in the Northeast region of Pennsylvania. After all filters were applied, there were a total of 121,057 patients with CT, MRI, and X-ray imaging and 22,348 patients with US imaging. The Institutional Review Board (IRB) approval was waived due to data being de-identified.

The SlicerDicer tool from Epic was used to extract data for the study. Data was extracted from a five-year period from the January 1, 2019 to the December 31, 2023. Epic (Epic Systems Corporation, Verona WI) is the electronic health record (EHR) software used by GHS. Patient charts within Epic contain provider notes, patient history and problem lists, medications, diagnostic test results, and a wide array of demographic information such as age, gender, and ethnicity. Individual charts were not accessed during the study such that any patient privacy concerns were alleviated. Epic is also used to determine billing for patients, and diagnoses require using either the International Classification of Diseases-10 (ICD-10) or Systematized Nomenclature of Medicine - Clinical Terms (SNOMED-CT) coding systems [7] to identify diagnoses. Stanson EDG [8] is a coding system that automatically groups similar ICD-10 codes using artificial intelligence. Diagnosis codes were utilized within the SlicerDicer tool to determine the number of patients meeting our criteria.

Based on our literature search, a set of 14 diagnoses or groups of diagnoses were identified. Diagnoses from the literature were identified in SlicerDicer by the research team according to ICD-10, SNOMED-CT, and Stanson EDG codes, then grouped by the research team in the following body regions: head and neck, upper extremity, lower extremity, thorax, abdomen, and pelvis. For all data collection, the date range was refined to between January 1, 2019 to December 31, 2023. The final step was to select the imaging study that was PoCUS-replaceable based on the literature and to select the imaging performed within the same encounter as the diagnosis. This gave us a cross-sectional result showing the number of patients who, for a given diagnosis, had undergone an imaging study in the same encounter as part of their diagnosis. For example, criteria were set to determine the number of patients with nephrolithiasis or hydronephrosis who had undergone a CT scan of the abdomen and pelvis in the same encounter that they received their diagnosis. Table 1 lists the diagnosis by ICD-10, Stanson EDG, and SNOMED-CT codes, as well as associated imaging. These codes were entered into SlicerDicer as part of the search process to identify specific diagnoses.

**Table 1:** Diagnoses, codes, and associated imaging. *There were no identifiable diagnosis codes with associated imaging that yielded usable data. ICD-10: International Classification of Diseases, 10th revision; SNOMED: Systematized Nomenclature of Medicine; MCL, medial collateral ligament; PCL, posterior cruciate ligament

Body Region	Diagnosis	Diagnosis Codes	Imaging Modality
Head and Neck	Pediatric Skull Fracture	ICD 10: S02	Head/Maxillofacial CT
	Cervical Spine Trauma	*	*
Upper Extremity	Pediatric Wrist/Forearm/Hand Fracture	SNOMED – Fracture at wrist and/or hand level	Wrist/Forearm X-ray
Lower Extremity	MCL/PCL/Medial Meniscus Injury	ICD-10: S83, M23	Knee MRI
	Adult Ankle Fracture	ICD-10: S82	Ankle X-ray
	Pneumothorax	ICD-10: J18.9, Stanson EDG pneumonia	Chest X-ray
	Pneumonia	ICD-10, J93, J95, S27.0, P25.1	Chest X-ray
	Hemothorax	ICD-10: J94.2, S27.1	Chest X-ray
	Pleural effusion	ICD-1o J90, J91	Chest X-ray
	Pericardial effusion	ICD-10: I131	Chest X-ray
Abdomen	Small Bowel Obstruction	SNOMED – Small Bowel Obstruction	CT Abdomen/Pelvis
	Diverticulitis	SNOMED – Diverticulitis and ICD K57	CT Abdomen/Pelvis
	Appendicitis	ICD-10: K35	CT Abdomen/Pelvis
	Abdominal Aortic Aneurysm	ICD: I71	CT Angiogram Abdomen/Pelvis
	Nephrolithiasis	Stanson EDG Kidney stones	CT Abdomen/Pelvis
	Hydronephrosis	ICD-10: N13, Q62, Stanson EDG Hydronephrosis	CT Abdomen/Pelvis
	Ovarian Torsion	ICD-10: N83	CT Abdomen/Pelvis
	Testicular Torsion	ICD-10: N44	CT Abdomen/Pelvis

Throughout the data collection process, several diagnoses were also found to have data regarding ultrasound for diagnostic use. Due to the limitations of SlicerDicer, it was unable to determine whether these ultrasound scans were point-of-care or departmental ultrasounds. Data from SlicerDicer regarding ultrasound use was concurrently collected for the diagnoses that had available data. Table 2 lists each diagnosis, its associated codes, and the type of ultrasound performed.

**Table 2:** Diagnoses and Codes for Ultrasound Imaging Data. ICD: International Classification of Diseases, revision 10

Diagnosis	Diagnosis Codes	Imaging Modality - Ultrasound
Appendicitis	ICD-10: K35	US Abdomen
Nephrolithiasis	Stanson EDG Kidney stones	US Renal
Hydronephrosis	ICD-10: N13, Q62, Stanson EDG Hydronephrosis	US Renal
Ovarian Torsion	ICD-10: N83	US Abdomen/Transvaginal
Testicular Torsion	ICD-10: N44	US Scrotum/Testes
Abdominal Aortic Aneurysm	ICD-10: I71	US Abdomen

## Results

**Figure 2:**
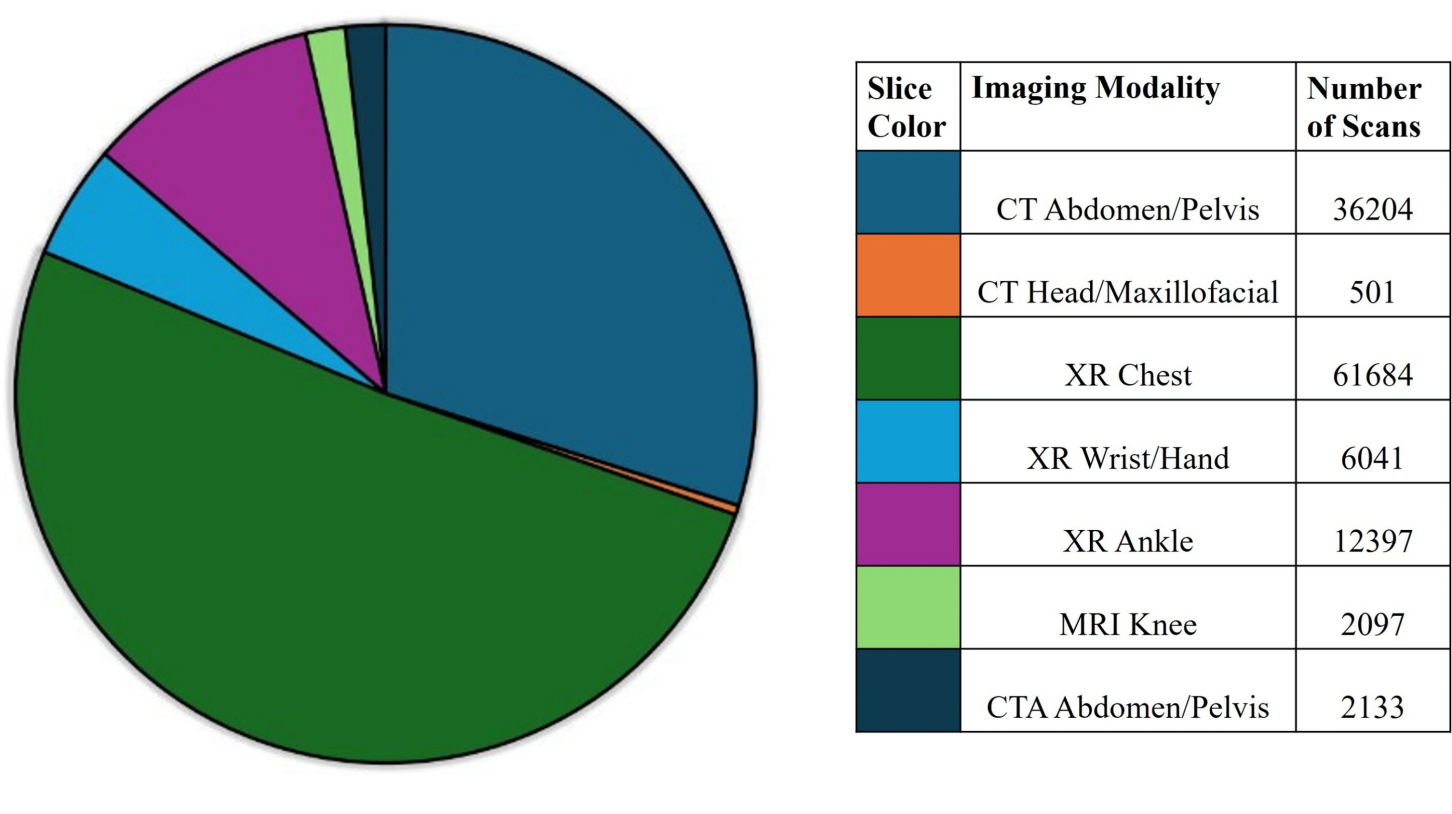
Totals number of cases by imaging modality Image credit: William Adams.

During the five-year study period of January 1, 2019 to December 31, 2023, a total of 121,057 scans reported on Epic were reviewed, which could have been replaced with PoCUS. These data were based on SlicerDicer search results. Figure 2 shows the total number of imaging scans by modality and body part. Of the 121,057 scans, 38,838 were CT scans. Of note, all scans aside from 2097 MRIs involved an imaging modality that exposes patients to ionizing radiation (e.g., CT and X-ray).

**Figure 3:**
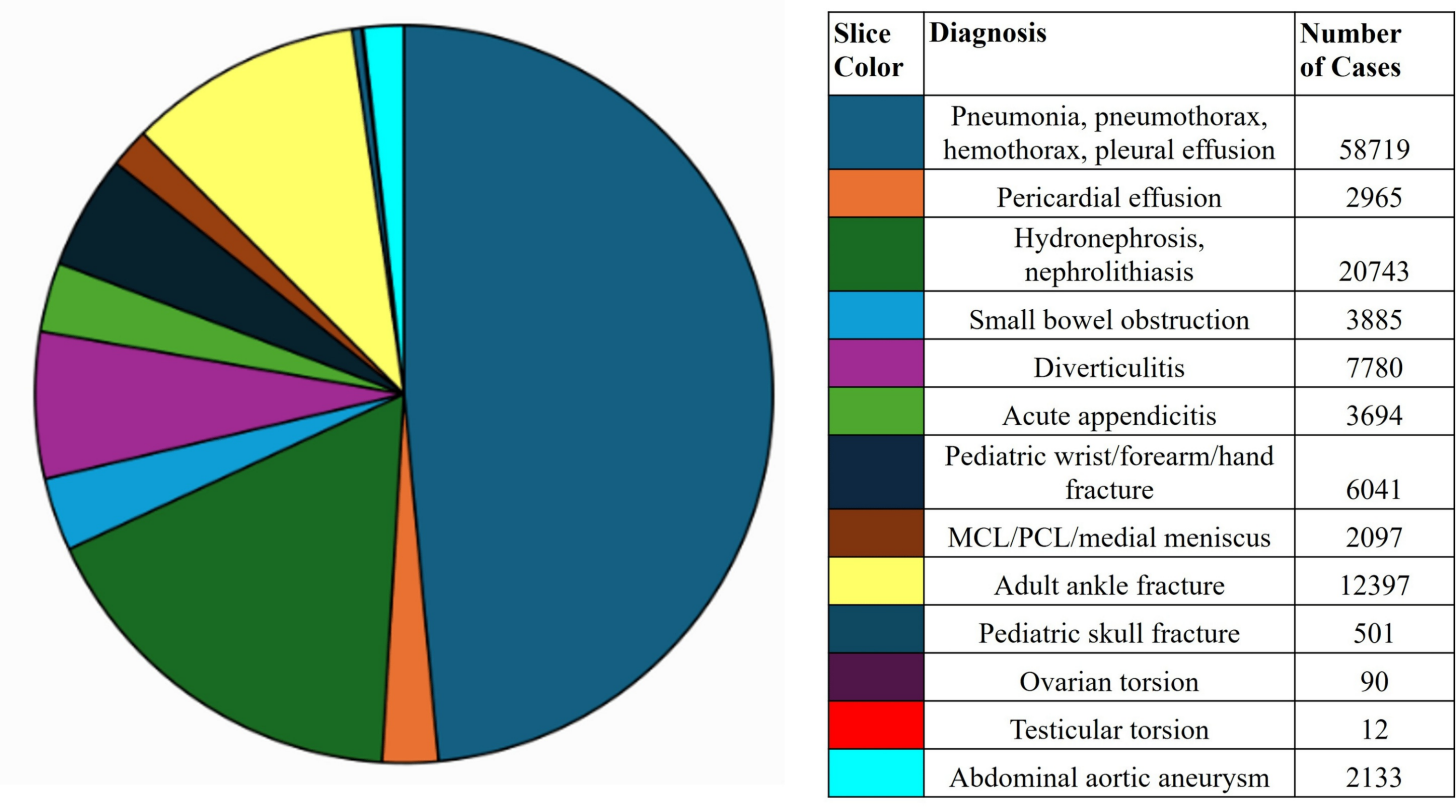
Number of patients by diagnosis. Image credit: William Adams.

Figure 3 shows the number of scans for each diagnosis. The largest single category was chest X-rays, with 61,684 scans performed for a variety of pulmonary and cardiac complaints for which PoCUS has proven to be accurate. These included pneumonia, pneumothorax, hemothorax, pleural effusion, and pericardial effusion. Most CT scans were performed for a variety of renal complaints, such as hydronephrosis and nephrolithiasis, with other large portions of CT scans being related to abdominal complaints, such as appendicitis, small bowel obstruction, and diverticulitis.

**Figure 4:**
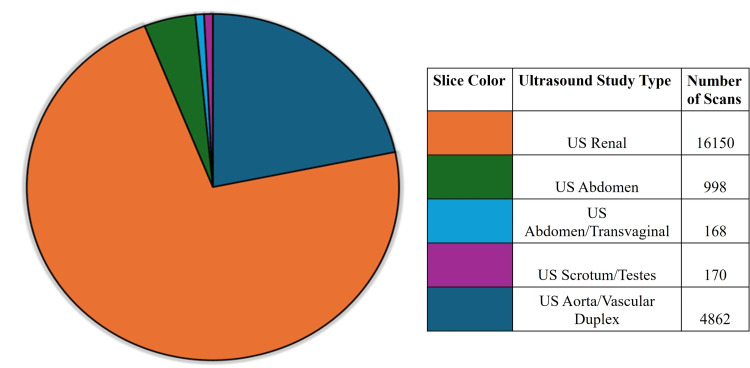
Number of patients screened by ultrasound based on body part. Image credit: William Adams.

Figure 4 displays the ultrasound (US) data, which were also collected when available. For each diagnosis, such as nephrolithiasis, SlicerDicer was checked for pertinent ultrasound scans used to diagnose the complaint. For renal complaints, 16,150 US scans were performed, compared to 20,743 CT scans performed for the same renal diagnoses during the same period. Other diagnoses made with US were testicular and ovarian torsion and abdominal aortic aneurysm (AAA). Unfortunately, SlicerDicer was unable to differentiate between PoCUS and radiology department ultrasound.

## Discussion

The authors are cognizant of the ethical considerations of using electronic health records (EHR) of patients that included the PoCUS information. Even when patient identifiers are removed, the risk of re-identifying patients remains, which could lead to privacy breaches. Maintaining strict safeguards to protect patient confidentiality at every stage is essential. Privacy is not just about removing names but ensuring that no combination of data points can be traced back to an individual, preserving their trust in the healthcare system [9].

Another significant concern is the potential misuse of this data by healthcare institutions and insurance companies. There is a fear that data might be leveraged to discriminate against certain groups, either by denying them necessary care or by making treatment decisions based on financial considerations rather than patient needs. This could lead to inequalities in the healthcare system, where some patients receive subpar care while others are overly scrutinized. To prevent these outcomes, it is crucial to establish clear ethical guidelines and involve all stakeholders - patients, providers, and policymakers - in conversations about how this data is used [9]. The goal should be to harness the power of PoCUS data for better care while safeguarding against practices that could harm patients.

Out of a total of 14 distinct diagnoses that were identified from 29 sources, 13 were included in the final data collection. Cervical spine injury did not yield any usable data from SlicerDicer. The following paragraphs highlight the uses of PoCUS based on diagnosis and standard imaging modality, as well as indications for use and additional benefits of using PoCUS. 

Head and neck regions

The use of PoCUS was supported for pathologies of the head and neck, such as skull fracture. Based on the data collected, sensitivities for skull fracture ranged from 81.8% to 92.3%, and specificities ranged from 95.9% to 100% [10,11]. In some instances, in addition to being fast and accurate, using PoCUS instead of a CT scan is less expensive and reduces exposure to ionizing radiation [11].

Upper and lower extremities

The diagnostic accuracy of PoCUS was also supported for pediatric forearm and wrist fractures, as well as lower extremity injuries such as ankle fractures and ligamentous knee injuries [12]. The use of point-of-care ultrasound for diagnosing wrist fractures, ankle fractures, and ligamentous knee injuries was highly sensitive and specific. A meta-analysis by Chartier reported 100% sensitivity and specificity of PoCUS for diagnosing wrist fractures and ankle fractures with PoCUS, and Stoianov similarly reported 100% sensitivity and specificity of PoCUS for ligamentous knee injuries [12,13]. This shows PoCUS to be a promising diagnostic tool for the detection of fractures and ligament injuries, especially in areas lacking access to other imaging modalities such as CT scans, or in cases of unavailable or malfunctioning X-ray machines.

Figure 5 from Ghosh et al. shows how PoCUS can detect medial collateral ligament (MCL) tears. The tissue disruption seen at the arrow indicates the torn ligament. MCL tears typically occur in the setting of direct trauma athletic injuries, and physical exam findings are essential but must be supplemented by objective imaging findings [14]. In these cases, PoCUS is more cost-effective and time-efficient than MRI and is comparable in diagnostic accuracy and usefulness in guiding patient treatment decisions [12].

**Figure 5:**
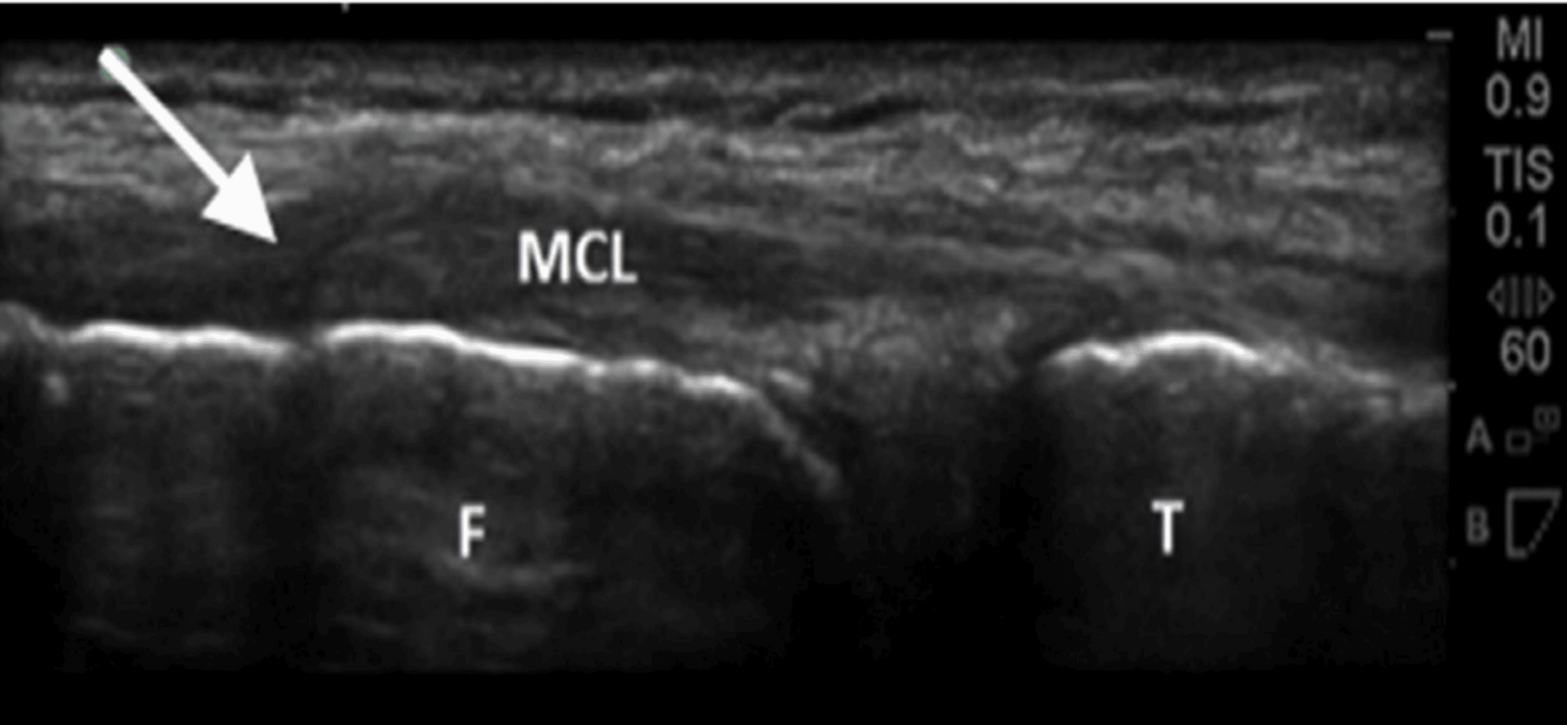
Ultrasound image of proximal MCL tear (arrow), noting ligament tissue disruption with retraction. Both the distal femur (F) and proximal tibia (T) are depicted with the adjoining MCL. The image is published under a Creative Commons License. From Ghosh et al. [14]. MCL: medial collateral ligament

Thoracic and abdominal regions

The findings by some of the authors indicate that PoCUS is effective in diagnosing pulmonary and cardiac pathologies such as pneumothorax [15], pneumonia [16], and pericardial [17] and pleural effusions [18]. DeMasi et al. investigated PoCUS for traumatic pneumothorax and reported 87% sensitivity and 100% specificity for clinically significant pneumothoraces that required tube thoracostomy [15]. Hanson and Chan reported faster diagnosis and treatment when patients underwent PoCUS scans versus other imaging modalities, including departmental echo, regarding pericardial effusions. This decreased delay suggests that PoCUS is superior to the traditional US in an emergent setting [17]. Toro et al. found that the sensitivity and specificity of PoCUS for community-acquired pneumonia ranged from 83% to 91.2% and 88% to 97.2%, respectively. The pertinent features of pneumonia observed on US imaging included air bronchogram, pleural effusion, b-lines, and subpleural consolidation. The authors highlighted that this is an operator-dependent technique and requires training and experience to ensure the accuracy of scans [16].

In addition to musculoskeletal and cardiothoracic complaints, PoCUS is effective in diagnosing abdominal complaints associated with appendicitis [19], small bowel obstruction [20], and diverticulitis [21]. Dumbrava and colleagues reported a sensitivity of 100% and a specificity of 44.4% for the detection of diverticulitis [21]. Abgottspon et al. showed PoCUS to be both sensitive and specific in diagnosing appendicitis in pregnant women. This use case is especially beneficial because it eliminates the dangers of radiation exposure for the developing fetus. The sensitivity of PoCUS was highest in the first trimester at 72.7%, and MRI is effective as a secondary tool where PoCUS is not definitive [22].

Pelvic region

**Figure 6:**
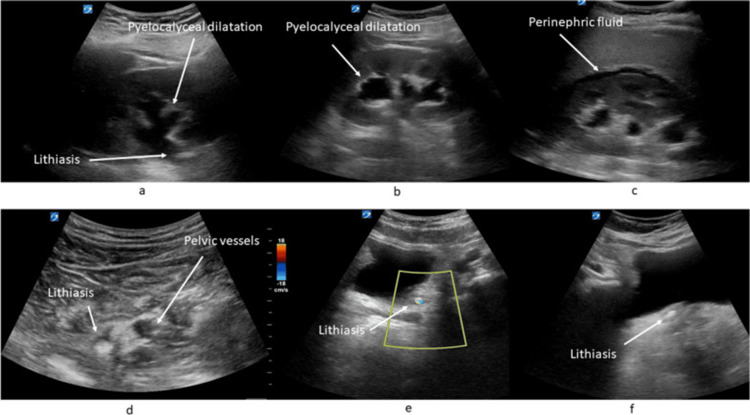
Various diagnoses made by PoCUS related to kidneys. “Ultrasound images: (a) pyelocalyceal dilatation and proximal lithiasis, (b) pyelocalyceal dilatation, (c) perinephric fluid, (d) pelvic lithiasis, (e) bladder lithiasis and twinkle artifact in color doppler; and (f) bladder lithiasis.” The image is published under a Creative Commons License. From Bourcier et al. [26].

For pelvic pathologies such as nephrolithiasis and testicular and ovarian torsion, PoCUS is a promising diagnostic tool [23-25]. Figure 6 displays examples of various diagnoses that can be made by PoCUS related to the kidneys. In the work of Bourcier et al., emergency physicians scanned the kidneys, ureters, and bladder along the two axillary lines and in the epigastric region. They assessed patients for pyelocalyceal dilatation, lithiasis in the bladder and proximal ureters, and perirenal effusion. Sensitivities were 95% for dilatation, 43% for lithiasis, and 57% for perinephric fluid [26].

Range of indications for PoCUS use

As the information above indicates, PoCUS can be used for many different diagnoses with varying levels of pre-test probability. With diagnoses such as pediatric wrist fractures where history and physical exam give high pre-test probability, PoCUS provides objective data to support the clinician’s gestalt. In children and adolescents with distal forearm injury, ultrasound was comparable to radiography concerning the outcome of function of the arm at four weeks [27]. Providers can also use PoCUS when a diagnosis is unclear. PoCUS can help with evaluating intra-abdominal free fluid and alternative traumatic abdominal pathology for diagnoses such as small bowel obstructions that could initially be missed with other imaging modalities [28]. An additional example is pericardial effusion, which only presents classically in 60% of cases. Many patients display non-specific symptoms of tachycardia, dyspnea, and peripheral edema. For these atypical presentations, PoCUS provides objective findings that can reorient the clinician and lead to faster diagnosis [17]. Overall, PoCUS can be helpful as both a confirmatory diagnostic method when clinicians are confident in their diagnosis and a rapid, low-cost investigative measure when clinicians seek to clarify an ambiguous diagnosis.

Additional benefits

Among the benefits of PoCUS are faster diagnosis and treatment, lower mortality, cost reduction, and decreased cancer risk from radiation exposure. In both Dumbrava et al. and Gibbons et al.’s respective reviews of PoCUS for diverticulitis and abdominal aortic aneurysm (AAA), respectively, decreases in time-to-diagnosis were noted for patients undergoing PoCUS versus standard CT imaging [21,29]. Similarly, Wang and colleagues measured length of stay (LOS) in emergency department patients with flank pain and found that early PoCUS (within 120 minutes) was associated with a significantly decreased LOS [30]. Hanson and Chan measured time-to-diagnosis and time-to-treatment for patients undergoing PoCUS versus a CT scan for pericardial effusion. The time-to-diagnosis was reduced by nearly 39 hours, and time-to-treatment was reduced by 21 hours when using PoCUS [17]. For AAA, Gibbons et al. also noted a significant decrease (20%-60%) in mortality when PoCUS is used instead of a CT scan. There are clear benefits to expedited diagnosis and treatment due to the emergent nature of AAA, which can rapidly progress and become fatal if it is not diagnosed and treated appropriately [29]. 

Brower and colleagues reviewed the diagnostic accuracy of PoCUS for small bowel obstruction. They considered the reduction in radiation exposure for patients undergoing PoCUS instead of CT scans. They projected the number of cancer cases (195) and deaths (98) that could be prevented by reducing radiation exposure. The review also projected $30 million in national cost savings by eliminating 143,000 CT scans by adopting a PoCUS-first approach for suspected small bowel obstruction [20]. 

Procedural uses of PoCUS

Our investigation of point-of-care ultrasound was limited to the diagnosis of pathologies. The use of PoCUS for procedures such as US-guided injections and fracture reductions was outside the scope of this work. Still, procedural uses of PoCUS were encountered extensively throughout the literature search process. Our literature review showed that ultrasound-guided procedures typically have better accuracy and efficacy than non-guided or fluoroscopy-guided procedures. PoCUS is frequently used to accurately administer intra-articular, intrabursal, peritendon, and perineural injections for diagnostic and therapeutic purposes [31]. PoCUS is also used in place of fluoroscopy to guide the reduction of forearm fractures and has been shown to lower patients’ radiation exposure, the number of attempts required for successful fracture reduction, and the risk of post-reduction compartment syndrome [12]. Exploring procedural uses of PoCUS is of interest to the research team and will be considered in future iterations of this work.

It seems that the use of PoCUS in GHS is not as pervasive as it could be, and there is a possibility for its application in a broad range of injuries and pathologies involving cardiopulmonary, gastrointestinal, renal, and musculoskeletal systems. For the diagnosis of AAA, hydronephrosis, appendicitis, and testicular and ovarian torsion, ultrasound imaging totals were available via SlicerDicer. It was not apparent whether these ultrasounds were point-of-care or radiology ultrasounds due to the limitations of information obtained from SlicerDicer, but the established use of ultrasound for these diagnoses may be promising for increased use of PoCUS within the health system.

Limitations

While the research team recommends the use of PoCUS where it is supported by the literature, it is important to consider the limitations of PoCUS. As stated by Toro and colleagues, PoCUS is an operator-dependent technique and requires specific training in order for clinicians to accurately diagnose pathologies [16]. Ultrasound also has difficulty in visualizing certain tissue types such as air-filled lung parenchyma [16] and any structure that is deep to the bony cortex, which cannot be penetrated by ultrasound waves [11]. Clinicians must consider these limitations when performing PoCUS scans, and patient safety should be of paramount concern when choosing an imaging modality.

## Conclusions

PoCUS is an imaging modality that allows for efficient diagnosis and treatment in the clinical space for inpatient, outpatient, and emergency providers. It can be used to diagnose abdominal and urogenital urgencies such as appendicitis, testicular torsion, ruptured ovarian cysts, and obstructive nephrolithiasis, as well as several non-emergent conditions involving the pulmonary and musculoskeletal systems. Based on the information collected and presented here, the Geisinger Health System and other institutions globally should increasingly utilize PoCUS as a primary diagnostic technique. By using this cost-effective, patient-centered, highly accurate bedside imaging technology where relevant, quick diagnoses can be made, and the negative side-effects of imaging modalities that involve radiation can be minimized, especially in patients with conditions where it is contraindicated. We further recommend that emergency medicine, primary care, sports medicine, and orthopedic clinicians unfamiliar with PoCUS undergo proper training, thereby taking advantage of this widely beneficial imaging modality.
